# Enhanced Resolution of DNA Separation Using Agarose Gel Electrophoresis Doped with Graphene Oxide

**DOI:** 10.1186/s11671-016-1609-0

**Published:** 2016-09-15

**Authors:** Jialiang Li, Yushi Yang, Zhou Mao, Wenjie Huang, Tong Qiu, Qingzhi Wu

**Affiliations:** 1 School of Chemical Engineering, Shandong University of Technology, Zibo, 255049 China; 2 State Key Laboratory of Advanced Technology for Materials Synthesis and Processing, Biomedical Material and Engineering Center, Wuhan University of Technology, Wuhan, 430070 China

**Keywords:** DNA fragments, Agarose gel electrophoresis, Graphene oxide

## Abstract

In this work, a novel agarose gel electrophoresis strategy has been developed for separation of DNA fragments by doping graphene oxide (GO) into agarose gel. The results show that the addition of GO into agarose gel significantly improved the separation resolution of DNA fragments by increasing the shift distances of both the single DNA fragments and the adjacent DNA fragments and completely eliminating the background noise derived from the diffusion of the excessive ethidium bromide (EB) dye in the gel after electrophoresis. The improved resolution of DNA fragments in GO-doped agarose gel could be attributed to the successive adsorption-desorption processes between DNA fragments and GO sheets, while the elimination of the background noise could be attributed to the adsorption of the excessive EB dye on the surface of GO sheets and high fluorescence quenching efficiency of GO. These results provide promising potential for graphene and its derivate utilized in various electrophoresis techniques for separation and detection of DAN fragments and other biomolecules.

## Background

Graphene has attracted considerable attention in biomedical fields due to its exceptional electronic, thermal, and mechanical properties, as well as extremely large specific surface area [1]. It is of great interest that graphene displays promising potential in DNA analysis and detecting [2–4]. The theoretical calculations indicate that DNA-graphene hybrids display significant base-dependent features in the electronic local density of states derived from the different interaction energies between DNA bases and graphene, providing an alternative route to DNA sequencing [5–10]. Studies showed that DNA fragments were quickly adsorbed on the surface of graphene oxide (GO) at room temperature due to the high affinity between GO and DNA nucleobases, while the adsorption and release of the double-stranded DNA from GO were relatively slow [3, 4]. A recent study showed that GO nanoplatelets were successfully utilized for extracting both DNA and RNA from eukaryotic and prokaryotic cells [11]. On the other hand, graphene and its derivates were reported as the super-quenchers with the long-range nanoscale energy transfer property [12–14]. Therefore, GO could bind and quench a dye-labeled single-stranded DNA probe and subsequently release the fluorescent probe when it formed a duplex with its target [15]. So far, various GO-based biosensors have also been extensively developed for DNA analysis with improved sensitivity and speed [9, 16–18]. For instance, a series of electrochemical biosensors with ultra-high resolution have been developed by depositing GO on the surface of graphite electrode for detection of DNA fragments at single-nucleotide base level and early diagnosis of leukemia (single abnormal cell in approximately 10^9^ normal cells) [19–21].

Agarose gel electrophoresis is one of the most important and routine techniques for DNA analysis. Combining with an organic dye (ethidium bromide (EB)), DNA fragments could be well separated according to the nucleobase amount and expediently observed under a UV light. The resolution of agarose gel electrophoresis for DNA separation is mainly dominated by the concentration of agarose gel and working voltage of electrophoresis. In most cases, dispersed and tailed DNA bands were obtained after electrophoresis, accompanying with serious background signals derived from EB dye. Therefore, it will be highly fascinating to develop a novel strategy to improve the electrophoresis resolution of DNA fractions with low-noise background.

**Scheme 1:**
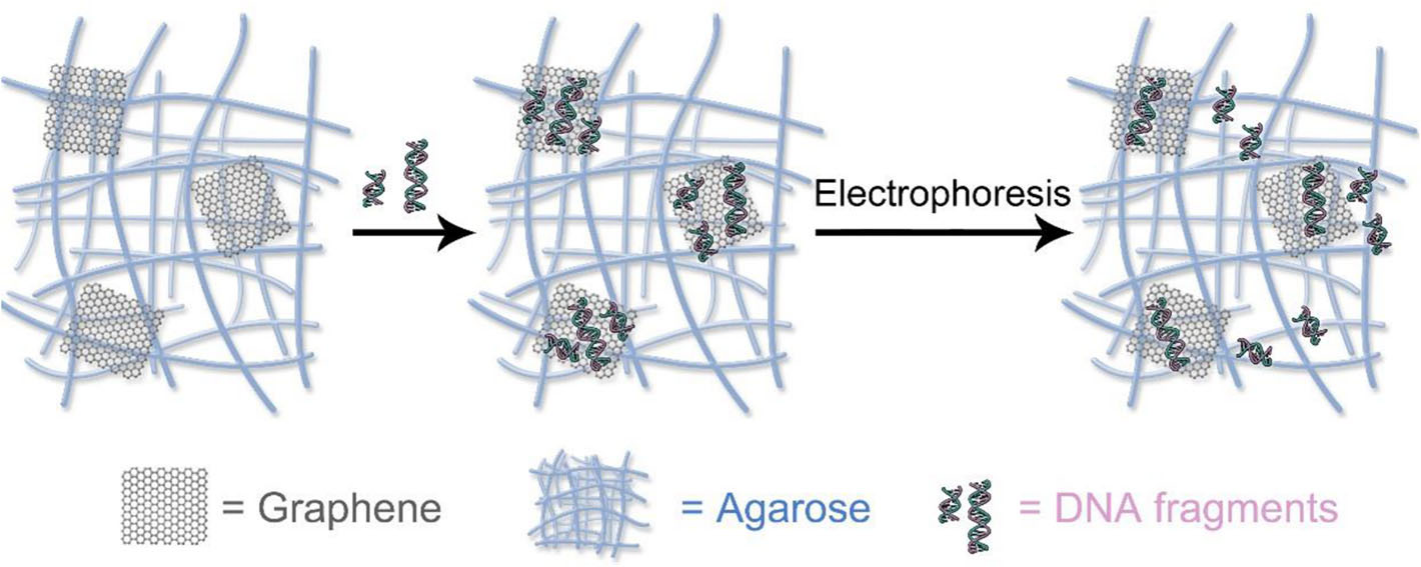
Illustration of GO-doped agarose gel electrophoresis for separation of DNA fragments

Herein, we report a novel electrophoresis strategy for DNA separation by adding GO into agarose gel. Compared with the routine agarose gel electrophoresis, successive adsorption-desorption processes between DNA fragments and the surfaces of GO nanosheets dispersed in the gel net significantly improved the separation of DNA fragments with different nucleobase amounts (Scheme 1). Meanwhile, the background noise derived from the diffusion of EB dye in the gel was completely eliminated because the excessive dye was adsorbed on the surface of GO nanosheets.

## Methods

### Reagents

Natural graphite powder, agarose, EB, and other chemicals were purchased from Sinopharm Chemical Reagent Corp. and used without further purification. Deionized water (16 MΩ cm) was obtained from a Nanopure Water Systems UV (Thomas Scientific, Swedesboro, NJ).

### Electrophoresis Separation of DNA Fragment

GO was prepared using natural graphite powder according to the modified Hummer’s method. In a typical DNA electrophoresis experiment, the as-prepared GO was dispersed in deionized water by ultrasonication (KQ2200E system, Kunshan Ultrasonic Instruments Co., Ltd, 40 KHz, 80 W) for 3 h. Then, the dispersed GO solution was added into agarose solution at designed concentrations and heated under microwave irradiation. DNA fragments containing standard DNA markers were separated using a DYCP-32A agarose horizontal electrophoresis system (Beijing Six One Instrument Corp.) at designed voltages and observed under a UV light. The shift distances and width of DNA bands were measured.

## Results and Discussion

**Fig. 1:**
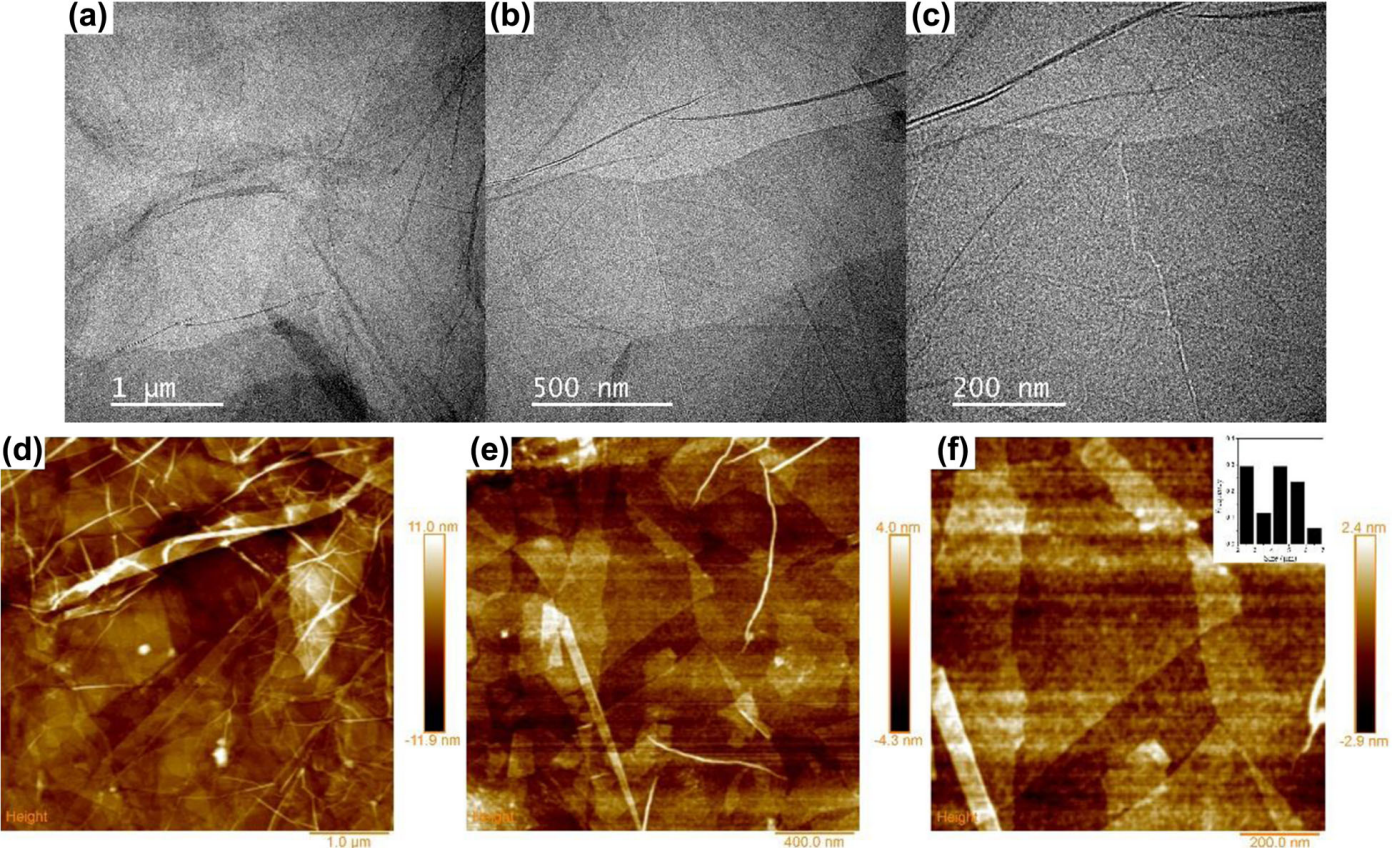
**a**–**e** TEM and AFM images of the as-synthesized GO nanosheets. The *inset* in the AFM image **f** shows the size distribution of the as-synthesized GO nanosheets

Figure 1 shows TEM and AFM images of the as-synthesized GO nanosheets. Numerous wrinkles were observed in the plane of the GO nanosheets (Fig. 1a–c). The AFM images show that inhomogeneous GO nanosheets were obtained in single- and multi-layers. The size of the GO nanosheets was calculated by measuring the area of the nanosheets and assuming it as a circle. The inset in Fig. 1f shows the lateral size distribution of the as-synthesized GO nanosheets, and the average size of the GO nanosheets was approximately 4.1 ± 1.3 μm.

**Fig. 2:**
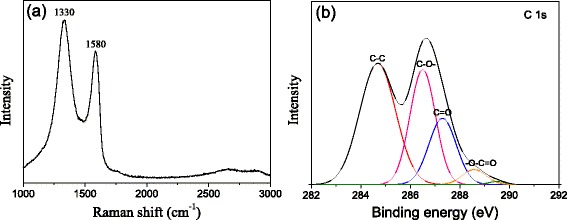
Raman and XPS spectra of the as-synthesized GO nanosheets

Figure 2 shows Raman and XPS spectra of the as-synthesized GO nanosheets. The characteristic peak at approximately 1580 cm^−1^ in the Raman spectrum (Fig. 2a) was assigned to the G band derived from the in-plane vibration of symmetric sp^2^ C–C bonds, while the peak at approximately 1330 cm^−1^ was derived from the first-order zone boundary phonons (D band). The chemical state of C element was analyzed through C 1s XPS spectrum (Fig. 2b). Four different carbon-bonding states were identified according to the peak fitting. The peaks at approximately 284.5, 286.6, 287.7, and 289 eV could be attributed to the C–C, C (epoxy)/C–OH, C=O, and O–C=O bonding configurations, respectively. In addition, the C/O atomic ratio calculated from the XPS spectrum was approximately 1.90, similar to that reported elsewhere [22].

**Fig. 3:**
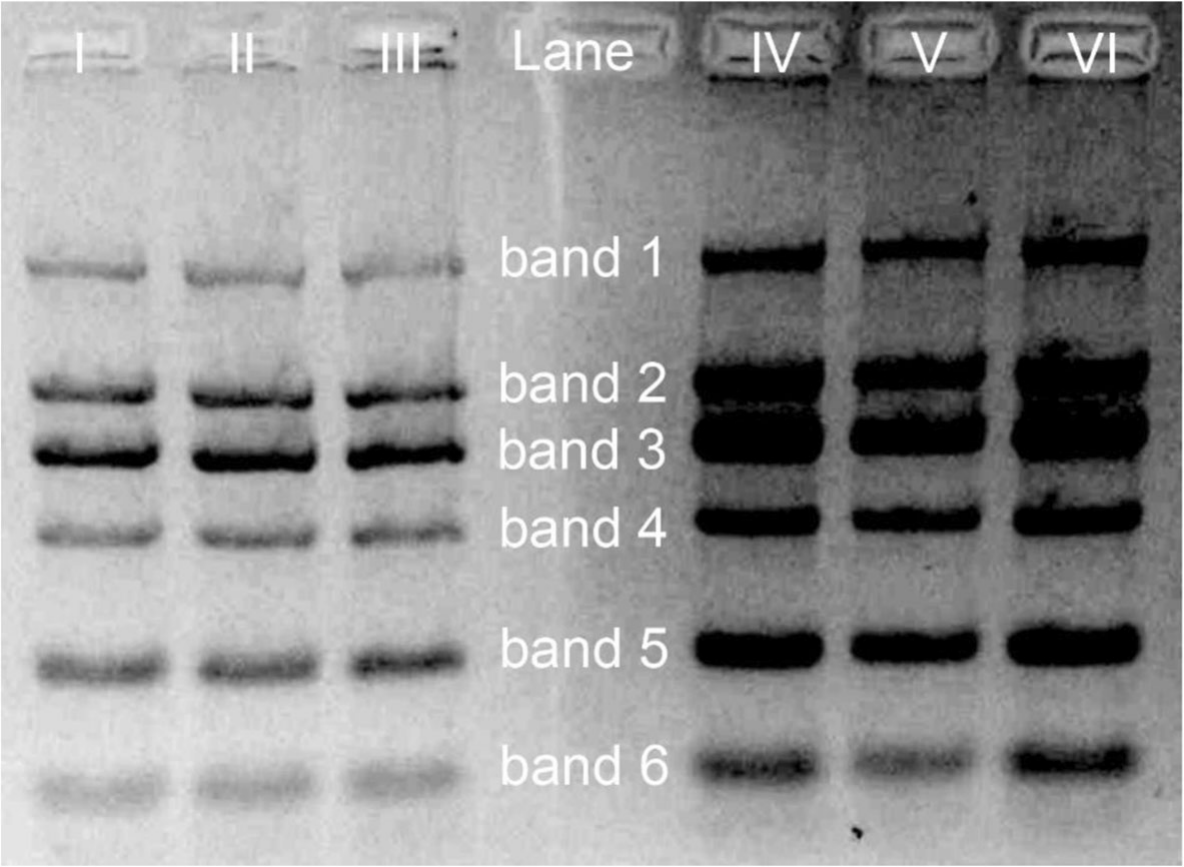
Agarose gel electrophoresis images of DNA fragments in the presence and absence of GO. *Lane I–III* photo image of agarose gel in the presence of GO, *Lane IV–VI* photo image of agarose gel in the absence of GO

Figure 3 shows the agarose gel electrophoresis image of a DNA sample with and without adding GO. In the presence of GO (lanes I–III), a clean gel was observed without EB-derived background noise. The elimination of EB-derived background noise in agarose gel could be attributed to the adsorption of EB dye on GO sheets. Moreover, the shift distances between different DNA bands were significantly enlarged, especially the shift between band 2 and band 3. In comparison, in the absence of GO (lanes IV–VI), the broad DNA bands in agarose gel were observed, accompanied with serious background noise throughout the gel due to the diffusion of EB dye in the gel. In particular, the shift distance between band 2 and band 3 was rather small.

**Fig. 4:**
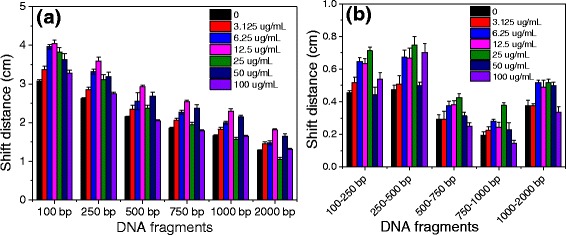
**a**, **b** Shift distances of DNA fragments after gel electrophoresis run at different concentrations of GO

It is generally known that the shift of DNA fragments in agarose gel was primarily depended on the nucleobase amount of DNA fragment and the voltage of electrophoresis. The short DNA fragments shift faster than the long fragments, and the shift rate of DNA fragments is promoted by increasing the voltage of electrophoresis. The influences of GO on the shift of DNA fragments in agarose gel were investigated by adjusting the concentration of GO in agarose gel. As shown in Fig. 4a, the shift distances of DNA fragments were negatively related with the length of DNA fragments regardless of adding GO in agarose gel. The addition of GO in agarose gel significantly increased the shift distance of DNA fragments compared with that in the absence of GO. The largest shift distance of DNA fragments was observed at the GO concentration of 12.5 μg/mL. The increased shift distance of DNA could be attributed to excellent conductivity of GO, promoting the electrophoresis rate of DNA fragments in agarose gel. However, the further increase of the GO concentration did not continually increase the shift distances of the DNA fragment. It is possible that the shift of DNA fragments slowed down in agarose gel due to the frequent adsorption-desorption processes between the DNA fragments and GO sheets when the concentration of GO exceeded 25 μg/mL. Figure 4b shows the influence of the GO concentrations on the shift distance between the two adjacent DNA fragments. With the increase of the GO concentration in agarose gel, the shift distances between the two adjacent DNA fragments were significantly increased, implying the better separation of DNA fragments. However, when the GO concentration in agarose gel increased up to 50 μg/mL, the shift distances between the two adjacent DNA fragments obviously decreased, indicating that the high concentration of GO in agarose gel hindered the separation of DNA fragments.

**Fig. 5:**
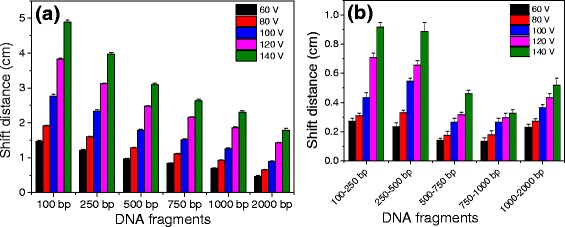
**a**, **b** Shift distances of DNA fragments after gel electrophoresis run at a GO concentration of 12.5 μg/mL under different voltages

Figure 5 shows the influence of electrophoresis voltages on the shift distances of DNA fragments in agarose gel at the GO concentration of 12.5 μg/mL. As shown in Fig. 5a, the shift distance of DNA fragments in agarose gel was positively related with the increase of electrophoresis voltages, indicating that the increase of electrophoresis voltages resulted in the increase of shift distances of DNA fragments. Figure 5b shows that the shift distances between the two adjacent DNA fragments increased with the enhancement of electrophoresis voltages, implying the better separation of DNA fragments.

Graphene and its derivate have attracted much attention for applications in DNA detection and sequencing due to its unique electronic property and single atom-layer thickness. The theoretical and experimental investigations demonstrated that single-nucleobase resolution of DNA sequencing could be realized by measuring the nucleobase-dependent transverse conductance derived from the translocation of DNA strands through the nanopores in the graphene plane [5–9]. Various biosensors for detection of DNA fragments and proteins have been developed based on either the extraordinarily high quenching efficiency of GO or the fluorescence resonance energy transfer between quantum dots and GO [12–18]. In addition, various biomolecules, including DNA, proteins, and peptides, were ready to be adsorbed on the surfaces of GO due to intramolecular interaction [10, 23]. The theoretical calculations and experimental measurement by isothermal titration calorimetry have demonstrated that the interaction energy of the DNA nucleobases with graphene was nucleobase-dependent with an order of guanine > adenine > thymine > cytosine, which was also affected by pH value of the solution [24, 25]. In the present study, DNA fragments were adsorbed onto the surfaces of GO nanosheets dispersed in agarose gel net by intramolecular interaction. The oxygen-containing functional groups in GO nanosheets could play a crucial role in the improvement of hydrogen-bonding interaction between DNA nucleobases and GO nanosheets, which is favorable to the adsorption of DNA fragments onto the surfaces of GO nanosheets. Subsequently, DNA fragments were desorbed from the surfaces of GO nanosheets under electrophoresis condition, which could be influenced by the charges carried on both the DNA fragments and GO nanosheets. Therefore, the successive adsorption-desorption processes between DNA fragments and GO nanosheets significantly improved the separation resolution of DNA fragments by increasing the shift distances between the adjacent DNA fragments with different nucleobase amounts. It is noticeable that reduced GO (rGO) nanosheets displayed size- and concentration-dependent cytotoxicity and genotoxicity in human mesenchymal stem cells, which was attributed to rGO-induced oxidative stress, cell membrane damage, DNA fragmentations, and chromosomal aberrations [26, 27]. However, in the present studies, there is no significant increase in both the amount and width of electrophoresis bands, confirming the absence of new DNA fragments derived from GO-induced fragmentations. Meanwhile, the background noise of the gel derived from the diffusion of the excessive EB dye in the gel disappeared because of the adsorption of EB dye molecules on the surfaces of GO nanosheets through the *π*-*π* interaction and the high fluorescence quenching efficiency of GO.

## Conclusions

In summary, a novel electrophoresis strategy has been developed for separation of DNA fragments using GO-doped agarose gel. The doping of GO in agarose gel resulted in the significant increase of the shift distances of both the single DNA fragment and the adjacent DNA fragments. The increased shift distance of DNA fragments could be attributed to excellent conductivity of GO, promoting the electrophoresis rate of DNA fragments in agarose gel. While the improved separation resolution for DNA fragments could be attributed to the successive adsorption-desorption processes between the surfaces of GO nanosheets dispersed in the gel net and DNA fragments with different nucleobase amounts, the background noise derived from the diffusion of EB dye in gel was completely vanished after electrophoresis due to the adsorption of the excessive EB dye by GO nanosheets. These results provide promising potential for graphene and its derivates utilized in various electrophoresis techniques for separation and detection of DNA fragments and other biomolecules.
